# The global and regional prevalence of oestrosis in sheep and goats: a systematic review of articles and meta-analysis

**DOI:** 10.1186/s13071-019-3597-2

**Published:** 2019-07-12

**Authors:** Md Ahaduzzaman

**Affiliations:** Department of Medicine & Surgery, Chattogram Veterinary & Animal Sciences University (CVASU), Chattogram, 4225 Bangladesh

**Keywords:** Epidemiology, Prevalence, Sheep bot fly, Nasal myiasis, Meta-analysis

## Abstract

**Background:**

Oestrosis, caused by the larvae of *Oestrus ovis*, commonly known as sheep nose bot, is an obligatory cavitary myiasis of sheep and goats. *Oestrus ovis* is a widespread parasite, but little is known about the prevalence of oestrosis at the global and broad geographical levels. The present study aimed to explore the epidemiology of oestrosis at the global and regional level to estimate prevalences and their associated factors using a systematic approach. This is, to the author’s knowledge, the first meta-analysis of oestrosis in sheep and goats.

**Methods:**

Published articles were obtained from nine electronic databases (PubMed, CAB Abstracts, Web of Science, Scopus, UCB library, Medline, Biosis Citation Index, Indian journals and Google Scholar) reporting the prevalence of *O. ovis* in sheep and goats from 1970 to 2018. Pooled prevalences were estimated using a random effect meta-analysis model.

**Results:**

Sixty-six studies were eligible, and data from 40,870 sheep and 18,216 goats were used for quantitative analysis. The random effect estimated prevalence of oestrosis at the global level in sheep was 51.15% (95% CI: 42.80–59.51%) and in goats was 42.19% (95% CI: 33.43–50.95%). The pooled prevalence estimates for Africa, Asia, Europe and the Americas were 47.85% (95% CI: 36.04–59.66%), 44.48% (95% CI: 33.09–55.87%), 56.83% (95% CI: 48.92–64.74%) and 34.46% (95% CI: 19.90–49.01%), respectively. Heterogeneity (*I*^2^ > 80%) was detected in most pooled estimates.

**Conclusions:**

Oestrosis is highly prevalent in many geographical regions of the world, especially in Europe and Africa. Factors that contribute to the pooled prevalence estimate of oestrosis need to be emphasised in any survey to estimate the true prevalence of oestrosis. Furthermore, there is a need for immunisation or implementation of other preventive measures to reduce the burden of oestrosis in sheep and goats and to improve the health and welfare status.

**Electronic supplementary material:**

The online version of this article (10.1186/s13071-019-3597-2) contains supplementary material, which is available to authorized users.

## Background

Oestrosis is a nasal myiasis, caused by the infestation of larvae of flies belonging to the genus *Oestrus* (Diptera: Oestridae) and is considered a severe parasitosis in sheep and goats and occasionally in other species of animals [1]. The adult female fly (*Oestrus ovis*) is commonly known as the sheep nasal bot fly which swarms around the heads of animals. The females are viviparous, and deposit previously hatched larvae (~ 500) directly to the nostril of host animals [2]. These larvae are obligatory parasites of nasal cavities and sinuses. The newly deposited first-stage larvae actively migrate to the nasal passage and attach to the mucous membranes [3]. Later they grow and undergo two moults to become third-stage larvae. During their migration and development, the larvae cause irritation and mechanical damage to the host nasal sinuses. The damaging effect to nasal sinuses can implicate acute clinical problems such as breathing difficulties, profuse nasal discharge and restlessness, which severely impairs the health of the affected animal [4]. Moreover, biomolecules secreted and excreted by larvae induce a local and systemic immune reaction that can exacerbate the situation [5, 6]. Mild infestation is typically asymptomatic but may induce signs of generalised disease including emaciation, which may lead to impaired animal production and consequently economic losses [7]. The infestation period is generally 25–30 days, but it can be longer (up to 10 months) depending on climatic conditions and other variables [3, 8]. The third-stage larvae are later expelled by the host sneezing onto the ground where they pupate and turn into adult flies. However, in some cases, the third-stage larvae fail to eject from the nasal sinuses and die. This may lead to septic sinusitis resulting in the death of animal [9]. The host response to oestrosis and larval burden are related to several factors such as susceptibility of host species, chronobiology of *O. ovis* at a particular geographical region and routine animal management practices [10]. Adult flies cause disturbances in flocks and substantial losses in animal production are particularly associated with the larval development of the parasite [11]. Adding to these issues, this parasite is difficult to control in the environment and there is a significant disparity in the therapeutic response of sheep and goats [12].

Oestrosis is primarily a myiasis of sheep and goats; however, there have been widespread reports of human infestation [13–15]. It is the most common cause of human ophthalmomyiasis and is typically occurs in shepherds and farmers [16]. Although it has also been reported in patients who have no association with animal husbandry and are far from any farming zone, the number of cases is limited [17, 18]. A high prevalence of oestrosis in sheep and goats in a geographical region may potentially increase the risk of zoonosis and may influence the occurrences of human infection.

Understanding the distribution of oestrosis and associated risk factors is essential to improving animal health. The disease is distributed worldwide and widespread in many regions of the world. An almost full-scale of prevalence estimates ranging between 5.88–100% of oestrosis in sheep and goats has been reported worldwide [19–23]. This inconsistent estimate in various geographical locations is plausible due to an aggregated distribution of the parasite in particular geographical areas and climatic conditions, methods for identifying the disease, the origin of the sample and sampling strategy, study duration and studied species of animal. An overview of knowledge on the geographical distribution and burden of oestrosis in sheep and goats will offer a better understanding of its impacts on animal production and prevent the spread of disease to humans. Therefore, this study aimed to estimate the global prevalence of oestrosis in sheep and goats and to assess the potential factors that contribute to the variability in the prevalence and distribution of the disease using a systematic approach.

## Methods

The study was conducted according to the guidelines provided by PRISMA (Preferred Reporting Items for Systematic Reviews and Meta-Analyses) for systemic review and meta-analysis. The PRISMA 2009 checklist (Additional file 1: Table S1) was followed to ensure the inclusion of relevant information and maintain study standard.

### Literature search

A systemic search strategy was used to identify all published articles reporting the prevalence of *O. ovis* in sheep and goats. Published works of literature were searched in nine electronic databases: PubMed, CAB Abstracts, Web of Science, Scopus, UCB library, Medline, Biosis Citation Index, Indian journals and Google Scholar, published between January 1970 and March 2018. Searches of the first seven listed databases were undertaken on 30th of March 2018, and the last two on 31st of March 2018. The search terms were categorised into descriptive, population and outcome as described before by Islam et al. [24]. The modified search terms are presented in Table 1. Search field option was selected as “All Fields”. The “descriptive term”, “population term” and “outcome term” were combined using the Boolean operator “AND”. Search terms were adjusted as per specification and minor differences in syntax rules of individual databases.

**Table 1:** Algorithm for electronic database search to find published reports on the prevalence of oestrosis in sheep and goats

Search term	Boolean keywords
Descriptive term	Prevalence OR incidence OR frequency OR occurrence OR detection OR identification OR isolation OR characterisation OR investigation OR survey OR rate
Population term	*Oestrus ovis* OR *O. ovis* OR sheep nasal bot OR sheep botfly OR bot fly larva OR nasal myiasis OR oestrosis OR estrosis OR oestrus myiasis OR *O. ovis* myiasis OR botfly encephalitis
Outcome term	Goat OR doe OR buck OR caprine OR ovine OR sheep OR ram OR ewe OR small ruminant

The search optimisation was performed for articles published in the English language. The reference list of all retrieved items was searched manually in triplicate to identify all eligible studies and to ensure that databases searches have missed no reports.

### Selection of studies

Articles were considered eligible for meta-analysis based on the following criteria: published between January 1970 and March 2018; full-text article; English language; any country of the world; reported as animal level prevalence data; studied population is sheep or goat or both; cross-sectional, case-control, longitudinal and cohort studies. Articles were excluded if prevalence data were not reported, species other than sheep or goat, case study, experimental trial and materials other than the English language.

### Quality of the studies

Selected studies were evaluated for quality of reporting and selection for bias using a quality appraisal checklist [25, 26] (Additional file 2: Text S1, Figure S1).

### Data extraction

The following data were extracted on a spreadsheet where possible from each eligible article: author, year of publication, country, region/province, continent, study duration, host, breed, origin of sample, method of detection/diagnosis, population, positive, prevalence, season when the prevalence was highest, ambient temperature during the peak prevalence season, specific risk (sex, age, coat colour), mean larval burden and the highest number of larvae per head. Overall, data from 59,086 animals (sheep and goats) of various geographical locations were analysed (Table 2).

**Table 2:** Characteristics of 66 studies included in this meta-analysis to investigate the pooled prevalence of oestrosis in sheep and goats

Reference	Year	Country	Study duration (months)	Host	Sample origin	Detection method	No. of samples	Positive *n* (%)
Benakhla et al. [20]	2004	Algeria	12	Sheep	Abattoir	Necropsy	313	211 (67.41)
Attindehou et al. [21]	2012	Benin	6	Sheep	Abattoir	Necropsy	256	90 (35.16)
				Goat	Abattoir	Necropsy	224	43 (19.20)
Amin et al. [22]	1997	Egypt	na	Sheep	Abattoir	Necropsy	1200	104 (8.67)
Osman [29]	2010	Egypt	12	Sheep	Abattoir	Necropsy	623	217 (34.83)
				Goat	Abattoir	Necropsy	357	83 (23.25)
Ramadan et al. [43]	2013	Egypt	12	Sheep	Abattoir	Necropsy	3132	360 (11.49)
Alem et al. [30]	2010	Ethiopia	5	Sheep	Abattoir	Necropsy	369	349 (94.58)
				Goat	Abattoir	Necropsy	431	381 (88.40)
Bekele et al. [44]	1995	Ethiopia	16	Sheep	Farm	Necropsy	376	23 (6.12)
Gebremedhin [31]	2011	Ethiopia	6	Sheep	Abattoir	Necropsy	311	217 (69.77)
				Goat	Abattoir	Necropsy	243	115 (47.33)
Yilma & Genet [32]	2000	Ethiopia	12	Sheep	Abattoir	Necropsy	248	192 (77.42)
				Goat	Abattoir	Necropsy	258	188 (72.87)
Gabaj et al. [33]	1993	Libya	5	Sheep	Abattoir	Necropsy	1489	336 (22.57)
				Goat	Abattoir	Necropsy	320	59 (18.44)
Negm-Eldin [34]	2015	Libya	12	Sheep	Abattoir	Necropsy	180	93 (51.67)
				Goat	Abattoir	Necropsy	120	34 (28.33)
Pandey & Ouhelli [45]	1984	Morocco	12	Sheep	Abattoir	Necropsy	120	83 (69.17)
Oniye [46]	2006	Nigeria	6	Sheep	Abattoir	Necropsy	116	72 (62.07)
Horak [47]	1977	South Africa	24	Sheep	Abattoir	Necropsy	542	398 (73.43)
Horak [1]	2005	South Africa	12	Sheep	Farm	Necropsy	193	103 (53.37)
				Goat	Farm	Necropsy	96	58 (60.42)
Pandey [48]	1989	Zimbabwe	13	Sheep	Abattoir	Necropsy	507	111 (21.89)
Biu & Nwosu [79]	1999	Nigeria	12	Goat	Abattoir	Necropsy	4000	2150 (53.75)
Horak & Butt [80]	1977	South Africa	13	Goat	Abattoir	Necropsy	130	96 (73.85)
Saleem et al. [49]	2017	India	12	Sheep	Abattoir	Necropsy	120	119 (99.17)
Sharma et al. [50]	2012	India	na	Sheep	Abattoir	Necropsy	128	25 (19.53)
Dhishonin et al. [51]	2017	India	12	Sheep	Abattoir	Necropsy	143	9 (6.29)
Jagannath et al. [35]	1989	India	12	Sheep	Abattoir	Necropsy	520	464 (89.23)
				Goat	Abattoir	Necropsy	263	219 (83.27)
Pathak [36]	1992	India	12	Sheep	Abattoir	Necropsy	384	312 (81.25)
				Goat	Abattoir	Necropsy	466	249 (53.43)
Dehghani et al. [37]	2012	Iran	6	Sheep	Abattoir	Necropsy	5934	1347 (22.70)
				Goat	Abattoir	Necropsy	1802	409 (22.70)
Shoorijeh et al. [38]	2010	Iran	13	Sheep	Abattoir	Necropsy	2002	994 (49.65)
				Goat	Abattoir	Necropsy	1998	261 (13.06)
Shoorijeh et al. [52]	2009	Iran	13	Sheep	Abattoir	Necropsy	2002	995 (49.70)
Tavassoli et al. [53]	2012	Iran	12	Sheep	Abattoir	Necropsy	402	122 (30.35)
AL-Ubeidi et al. [54]	2017	Iraq	3	Sheep	Abattoir	Necropsy	133	72 (54.14)
Abo-Shehada et al. [55]	2000	Jordan	17	Sheep	Abattoir	Necropsy	417	242 (58.03)
Othman [56]	2009	Palestine	12	Sheep	Abattoir	Necropsy	335	181 (54.03)
Alahmed [19]	2000	Saudi Arabia	12	Sheep	Abattoir	Necropsy	544	32 (5.88)
Alikhan et al. [57]	2018	Saudi Arabia	na	Sheep	Abattoir	Necropsy	1334	400 (29.99)
Hanan [58]	2013	Saudi Arabia	12	Sheep	Abattoir	Necropsy	480	257 (53.54)
Arslan et al. [59]	2009	Turkey	12	Sheep	Abattoir	Necropsy	387	156 (40.31)
Ipek & Altan [39]	2017	Turkey	2	Sheep	Abattoir	Semi-nested PCR	158	133 (84.18)
				Goat	Abattoir		26	10 (38.46)
				Sheep	Abattoir	Rhinoscopy	158	104 (65.82)
				Goat	Abattoir		26	10 (38.46)
Karatepe et al. [60]	2014	Turkey	12	Sheep	Abattoir	Necropsy	364	82 (22.53)
Özdal et al. [61]	2016	Turkey	12	Sheep	Abattoir	Necropsy	328	127 (38.72)
Uslu & Dik [62]	2006	Turkey	13	Sheep	Abattoir	Necropsy	624	368 (58.97)
Rahman & Karim [81]	1989	Bangladesh	12	Goat	Abattoir	Necropsy	705	175 (24.82)
Huq [82]	1983	Bangladesh	15	Goat	Abattoir	Necropsy	600	114 (19.00)
Jumde & Dixit [83]	2012	India	na	Goat	Abattoir	Necropsy	247	194 (78.54)
Shoorijeh et al. [84]	2011	Iran	13	Goat	Abattoir	Necropsy	1998	261 (13.06)
Abo-Shehada et al. [85]	2003	Jordan	13	Goat	Abattoir	Necropsy	520	126 (24.23)
Dorchies et al. [40]	2000	France	12	Sheep	Abattoir	Necropsy	631	274 (43.42)
				Goat	Abattoir	Necropsy	672	191 (28.42)
Yilma & Dorchies [63]	1991	France	12	Sheep	Abattoir	Necropsy	555	361 (65.05)
Bauer et al. [64]	2002	Germany	24	Sheep	Farm	ELISA	1497	753 (50.30)
Papadopoulos et al. [7]	2006	Greece	na	Sheep	Mixed flock	ELISA	397	193 (48.61)
				Goat	Mixed flock	ELISA	335	58 (17.31)
Papadopoulos et al. [23]	2001	Greece	12	Sheep	Mixed flock	ELISA	300	300 (100)
				Goat	Mixed flock	ELISA	500	212 (42.40)
Papadopoulos et al. [41]	2010	Greece	12	Sheep	Abattoir	Necropsy	292	126 (43.15)
				Goat	Abattoir	Necropsy	158	120 (75.95)
Caracappa et al. [65]	2000	Italy	24	Sheep	Abattoir	Necropsy	841	469 (55.77)
Scala et al. [66]	2001	Italy	12	Sheep	Free-ranging flocks	Necropsy	566	514 (90.81)
Scala et al. [3]	2002	Italy	12	Sheep	Abattoir	ELISA and necropsy	443	327 (73.81)
Cozma et al. [67]	2010	Romania	6	Sheep	Farm	Necropsy and skin sensitivity test	280	140 (50.00)
Daniela [42]	2008	Romania	9	Sheep	Mixed flock	Necropsy	84	57 (67.86)
				Goat	Mixed flock	Necropsy	51	22 (43.14)
Alcaide et al. [68]	2005	Spain	9	Sheep	Farm	ELISA and necropsy	276	218 (78.99)
Alcaide et al. [69]	2005	Spain	23	Sheep	Farm	ELISA	5878	4070 (69.24)
Gracia et al. [70]	2010	Spain	12	Sheep	Abattoir	Necropsy	120	101 (84.17)
Gracia et al. [71]	2006	Spain	na	Sheep	Pasture and indoor	Necropsy	20	14 (70.00)
Paredes-Esquivel et al. [72]	2009	Spain	2	Sheep	Free ranging flocks	Necropsy	206	173 (83.98)
Paredes-Esquivel et al. [73]	2012	Spain	13	Sheep	Abattoir	Necropsy	554	255 (46.03)
Alcaide et al. [86]	2005	Spain	23	Goat	Farm	ELISA	1590	717 (45.09)
			9	Goat	Farm	Necropsy	80	28 (35)
Carvalho et al. [74]	2015	Brazil	80	Sheep	Farm	Necropsy	71	12 (16.90)
Silva et al. [75]	2013	Brazil	8	Sheep	Abattoir	Necropsy	139	19 (13.67)
Silva et al. [76]	2012	Brazil	36	Sheep	Farm	Necropsy	72	36 (50.00)
Hidalgo et al. [77]	2015	Chile	4	Sheep	Abattoir	Necropsy	87	53 (60.92)
Murguía et al. [78]	2000	Mexico	na	Sheep	Farm	Thin layer immune assay test	689	229 (33.24)

*Abbreviations*: ELISA, enzyme-linked immunosorbent assay; na, not mentioned

### Data analysis

All obtained data were entered and sorted in a Microsoft Excel spreadsheet. Prevalence was estimated by the number of positive animals divided by the total number of animals. Only the crude estimate of prevalence was used and their 95% confidence interval (CI). The CI was calculated using the standard formula for a proportion (p): ${\text{p}}\, \pm \, 1.96\sqrt {\left[ {{\text{p}}\, \times \,\left( {100\, - \,{\text{p}}} \right)\, \div \,n} \right]}$, where *n* is the studied population size [24]. In circumstances where the higher limit of CI exceeded 100, the value was settled to 100 to avoid > 100% prevalence. Data were analysed using STATA v.11.0 (StataCorp LP, College Station, TX, USA). The meta-analysis was performed using the STATA command “metan”. The percentage of heterogeneity across studies that is due to variation rather than chance was estimated by interpreting the *I*^2^ statistic value and Cochran’s *Q* (represented as *χ*^2^ and *P*-values) [27]. The *I*^2^ values of 25, 50 and 75% were considered as low, moderate and high heterogeneity, respectively [27]. Due to a high degree of heterogeneity between studies the random effect model was selected for summary statistics. Furthermore, the potential sources of heterogeneity were investigated by subgroup analysis. Five potential sources of heterogeneity were examined: continent, country, species, origin of the sample and the method of detection. For constructing a forest plot, data of each continent were analysed separately with the stratified command “by” for the variables. The results are presented as prevalence percentage with 95% CI. Assessment of small study effects was determined using two funnel plots, and the sources of funnel plots asymmetry were also tested to identify the publication bias by Egger’s test [28].

Additionally, analysis of climatic data was performed by extracting the ambient temperature data during the peak prevalence season directly from the prospective articles or, where the peak prevalence season was reported but temperature was not mentioned, from the national weather databases. Only the crude estimate of average temperature was used and their minimum temperature (Tmin) and maximum temperature (Tmax). Average ambient temperature was calculated using the formula: (Tmax + Tmin)/2. Similar to other variables, due to a high degree of heterogeneity the random effect model was selected for summary statistics. In circumstances where the average ambient temperature value was a proportion, the value was rounded to the nearest number using the ceiling and floor functions of Microsoft Excel 2016. Three potential sources of heterogeneity were examined: continent, country and temperature range. For constricting the forest plot, data of continent, country and temperature sub-group were analysed separately. The results are presented as degree Celsius (°C) with 95% CI of the mean.

## Results

### Search results and eligible studies

**Fig. 1:**
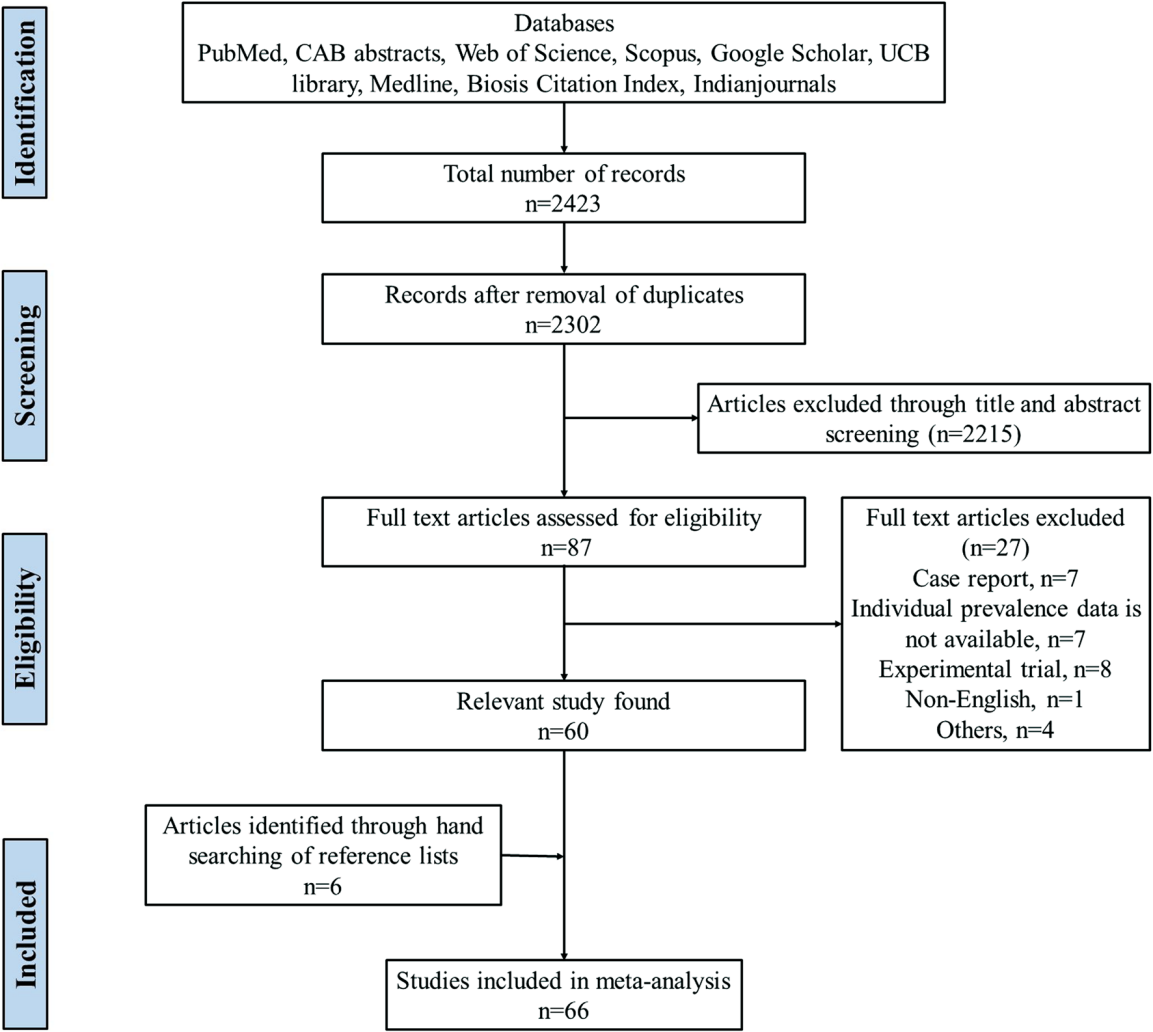
Flow diagram of the selection of eligible studies for inclusion in the meta-analysis

Figure 1 shows the search results. In the initial search on selected databases, 2423 potential articles were identified. After screening, a total of 87 eligible articles were found of which 27 articles were excluded due to following reasons: case report (*n* = 7); individual prevalence data not available (*n* = 7); experimental trial (*n* = 8); article other than the English language (*n* = 1); and others (*n* = 4). The list of excluded articles along with reasons for their exclusion is provided in Additional file 3: Text S2. A total of 66 eligible articles were used for meta-analysis (Additional file 4: Text S3). Among the selected articles, 18 articles reported the prevalence of oestrosis in both sheep and goats [1, 7, 21, 23, 29–42], 40 articles in sheep [3, 19, 20, 22, 43–78] and 8 articles reported only in goats [79–86]. Based on the origin of samples, 50 studies were from abattoirs [3, 19–22, 29–41, 43, 45–63, 65, 70, 73, 75, 77, 79–85], 10 from farms [1, 44, 64, 67–69, 74, 76, 78, 86], 3 from free ranging flocks [66, 71, 72] and 3 from mixed flocks [7, 23, 42]. Based on the method of diagnosis, 56 studies used necropsy [1, 19–22, 29–38, 40–63, 65, 66, 70–77, 79–85], 4 used ELISA [7, 23, 64, 69] and 6 used combined or other methods [3, 39, 67, 68, 78, 86]. A description of the characteristics of each included study is shown in Table 2.

### Continents and countries

All included studies on sheep and goats represent data from 5 continents, covering 26 countries of the world. The highest number of articles (*n* = 25) were from Asia covering 8 countries: India (*n* = 6), Iran (*n* = 5), Turkey (*n* = 5), Saudi Arabia (*n* = 3), Bangladesh (*n* = 2), Jordan (*n* = 2), Iraq (*n* = 1) and Palestine (*n* = 1). The second highest was from both Africa and Europe, with the same number of articles from each continent (*n* = 18). Countries within Africa contributed as follows: Ethiopia (*n* = 4), Egypt (*n* = 3), South Africa (*n* = 3), Libya (*n* = 2), Nigeria (*n* = 2), Algeria (*n *= 1), Benin (*n* = 1), Morocco (*n* = 1) and Zimbabwe (*n* = 1). European countries reported as follows: Spain (*n* = 7), Greece (*n* = 3), Italy (*n* = 3), France (*n* = 2), Romania (*n* = 2) and Germany (*n* = 1). Four articles were from South America: Brazil (*n* = 3) and Chile (*n* = 1). One article was from North America: Mexico (*n* = 1).

### Prevalence estimates

**Fig. 2:**
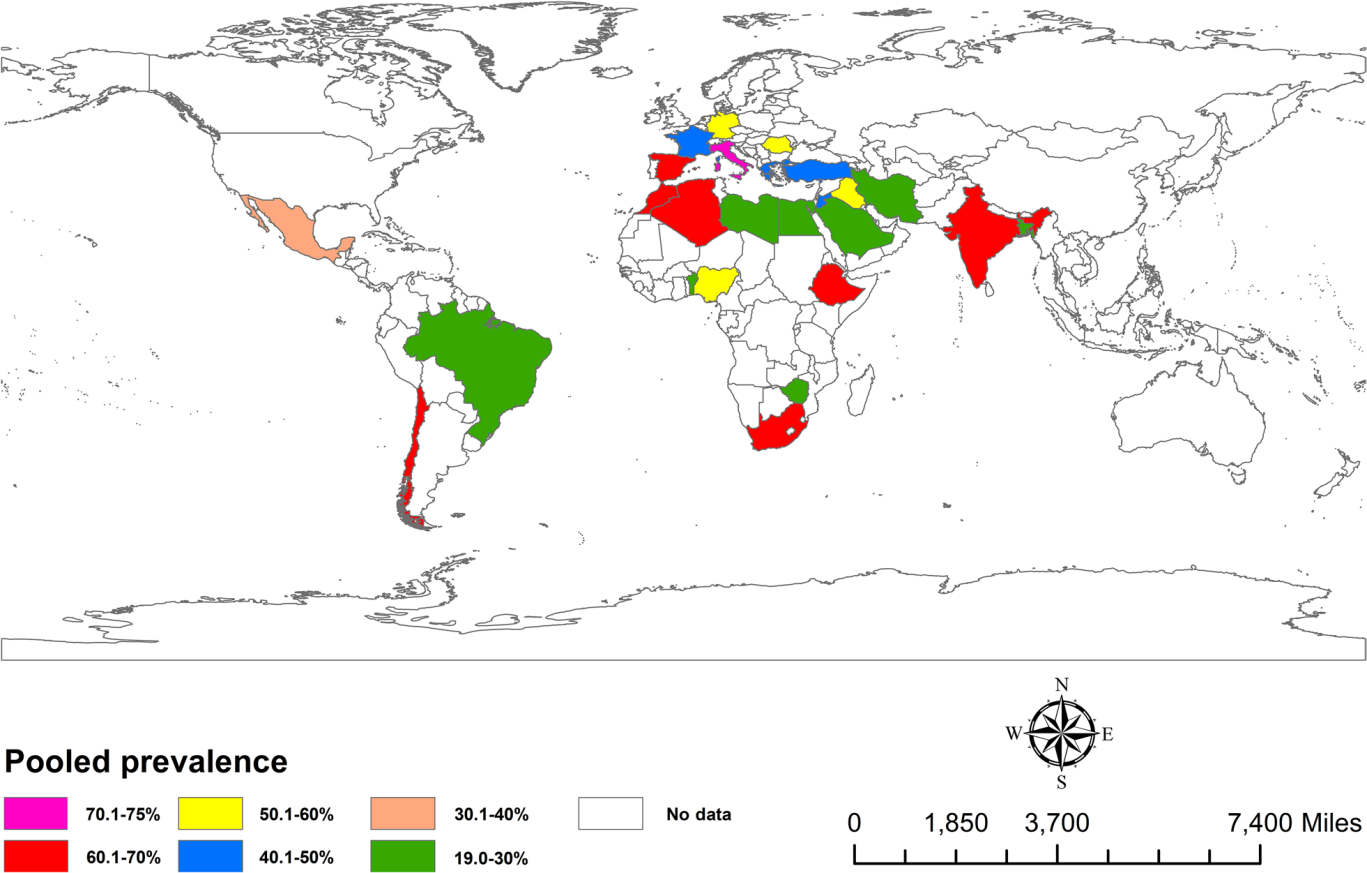
Estimated prevalence of oestrosis caused by *Oestrus ovis* in sheep and goats in different countries of the world from 1970 to 2018. The prevalence estimate is based on a meta-analysis of 66 studies comprising 59,086 sheep and goats. The map was produced using ArcGIS v.10.3.1 (Esri, Redlands, CA, USA)

On the basis of the global burden of oestrosis in sheep, the estimated prevalence ranged from 34.45% (95% CI: 19.90–49.01%) to 63.69% (95% CI: 56.08–71.30%) with considerable heterogeneity (*I*^2^= 96.7%, *P* < 0.0001). The random effect estimated global prevalence in sheep was 51.15% (95% CI: 42.80–59.51%) (Table 3). Likewise, the estimated prevalence of oestrosis in goats ranged from 37.01% (95% CI: 25.91–48.11%) to 48.56% (95% CI: 33.04–64.09%) with high heterogeneity (*I*^2^= 99.4%, *P* < 0.0001). The random effect estimated global prevalence in goats was 42.19% (95% CI: 33.43–50.95%) (Table 4). Overall, the global pooled estimated prevalence of oestrosis in sheep and goats was 48.25% (95% CI: 41.82–54.67%) with substantial heterogeneity (*I*^2^= 99.7%, *P* < 0.0001) (Table 5). The global estimated pooled prevalence of oestrosis in sheep and goats by country are shown in Fig. 2.

**Table 3:** Estimated pooled prevalence of oestrosis in sheep by world region

World region	No. of studies	No. of sheep sampled	No. of positive sheep	Pooled estimate %	95% CI	Heterogeneity (*χ*^2^)	*I*^2^ (%)	*P*-value
Global estimate	58	40,870	18,194	51.15	42.80–59.51	26,703.21	99.8	< 0.0001
Africa	16	9975	2959	47.41	32.16–62.65	6591.58	99.8	< 0.0001
Asia	20	16,897	6541	48.28	33.04–63.53	12,879.06	99.8	< 0.0001
Europe	17	12,940	8345	63.69	56.08–71.30	1108.77	98.6	< 0.0001
North and South America	5	1058	349	34.45	19.90–49.01	76.80	94.8	< 0.0001

*Abbreviations*: CI, confidence interval; *χ*^2^, Cochran’s *Q* Chi square; *I*^2^, inverse variance index

**Table 4:** Estimated pooled prevalence of oestrosis in goats by world region

World region	No. of studies	No. of goats sampled	No. of positive goats	Pooled estimate %	95% CI	Heterogeneity (*χ*^2^)	*I*^2^ (%)	*P*-value
Global estimate	26	18,216	6583	42.19	33.43–50.95	5277.62	99.5	< 0.0001
Africa	10	6179	3207	48.56	33.04–64.09	1247.18	99.3	< 0.0001
Asia	10	8651	2028	37.01	25.91–48.11	1656.08	99.4	< 0.0001
Europe	6	3386	1348	40.93	28.93–52.92	291.83	97.9	< 0.0001
North and South America^a^	0	–	–	–	–	–	–	–

^a^No records of goat oestrosis from North or South America

*Abbreviations*: CI, confidence interval; *χ*^2^, Cochran’s *Q* Chi square; *I*^2^, inverse variance index

**Table 5:** Pooled prevalences and estimated sources of heterogeneity in the prevalence of oestrosis in sheep and goats

Variable	Population	Pooled estimate prevalence (%)	95% CI	Heterogeneity (*χ*^2^)	*I*^2^ (%)	*P*-value
World region						
Global estimate	59,086	48.25	41.82–54.67	33,292.86	99.7	< 0.0001
Africa	16,154	47.85	36.04–59.66	9371.58	99.7	< 0.0001
Asia	25,548	44.48	33.09–55.87	17,688.62	99.8	< 0.0001
Europe	16,326	56.83	48.92–64.74	2380.45	99.1	< 0.0001
North and South America	1058	34.46	19.90–49.01	89.80	95.5	< 0.0001
Age						
Young (≤ 1 year)	18,188	38.30	27.21–49.38	8266.67	99.8	< 0.0001
Adult (> 1 year)	18,188	49.53	38.02–61.03	7006.91	99.7	< 0.0001
Sex						
Male	12,533	39.55	27.73–51.36	2288.98	99.6	< 0.0001
Female	12,533	48.74	31.16–66.33	6151.18	99.8	< 0.0001
Origin of sample						
Abattoir	49,124	47.72	40.53–44.90	29,650.45	99.8	< 0.0001
Farm	9962	50.78	36.49–65.07	2658.55	99.5	< 0.0001
Method of detection						
Necropsy	46,398	47.16	39.81–54.51	29,999.03	99.8	< 0.0001
Serology	11,075	50.87	39.09–62.65	995.73	99.2	< 0.0001
Other	1613	56.18	37.80–74.57	360.69	98.3	< 0.0001
Study duration (months)						
≤ 6	12,200	49.19	37.73–60.64	3046.99	99.4	< 0.0001
> 6 to ≤ 12	19,908	52.23	40.57–63.89	18,701.79	99.8	< 0.0001
> 12	22,219	41.78	30.37–53.19	6898.52	99.7	< 0.0001

*Abbreviations*: CI, confidence interval; *χ*^2^, Cochran’s *Q* Chi square; *I*^2^, inverse variance index

**Fig. 3:**
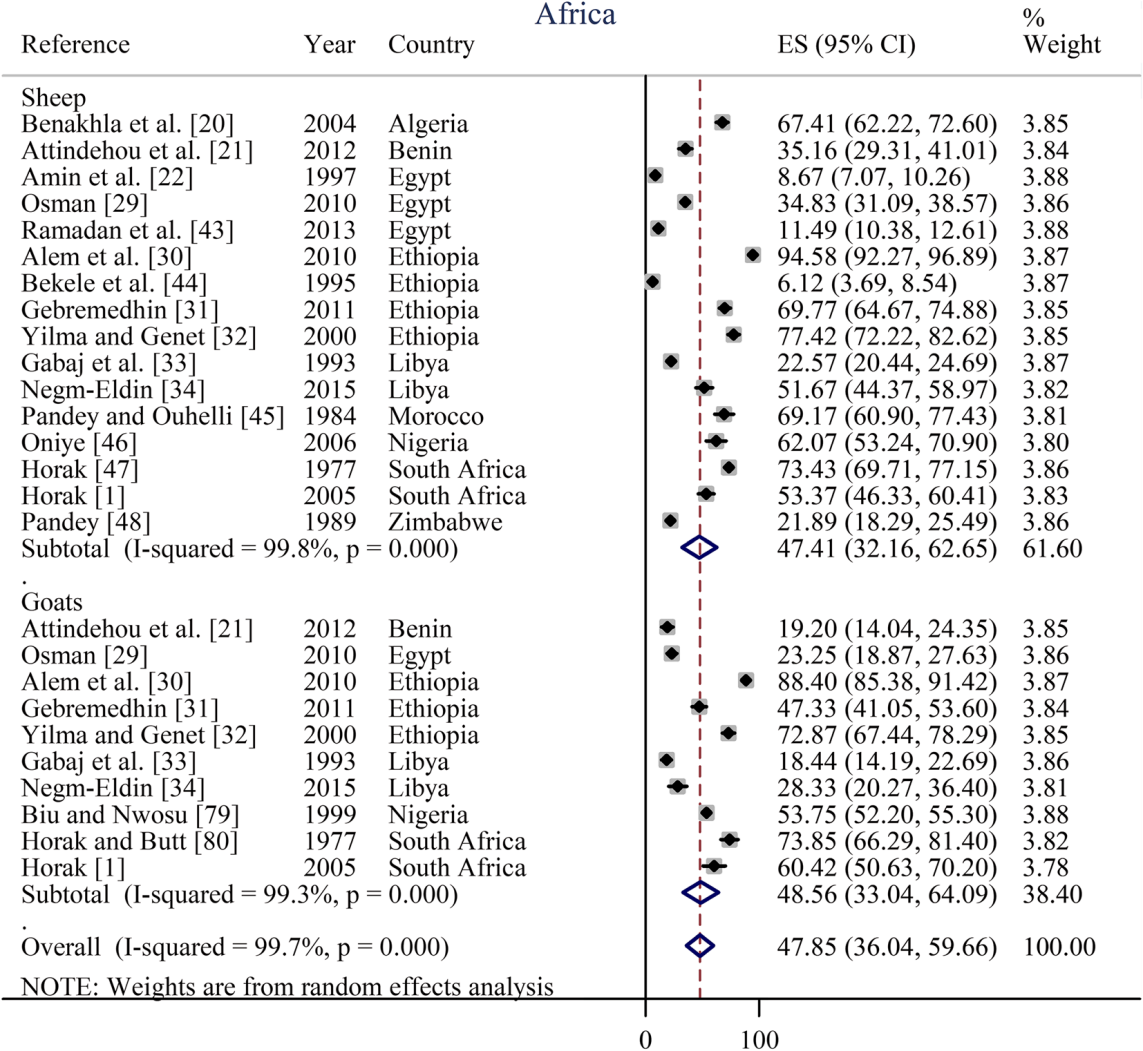
Forest plot of the prevalence estimates of oestrosis caused by *Oestrus ovis* in sheep and goats amongst studies conducted in Africa

**Fig. 4:**
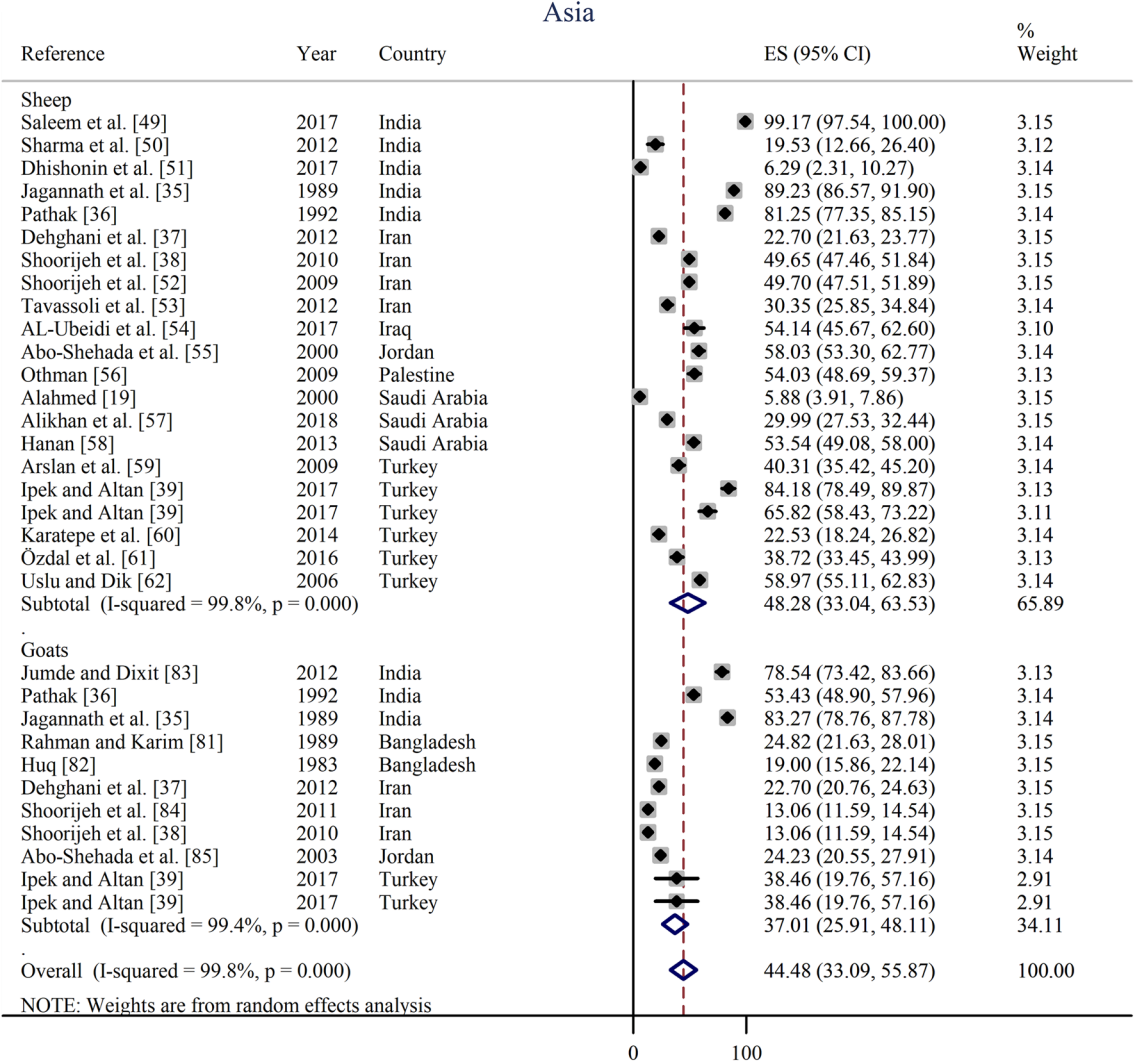
Forest plot of the prevalence estimates of oestrosis caused by *Oestrus ovis* in sheep and goats amongst studies conducted in Asia

**Fig. 5:**
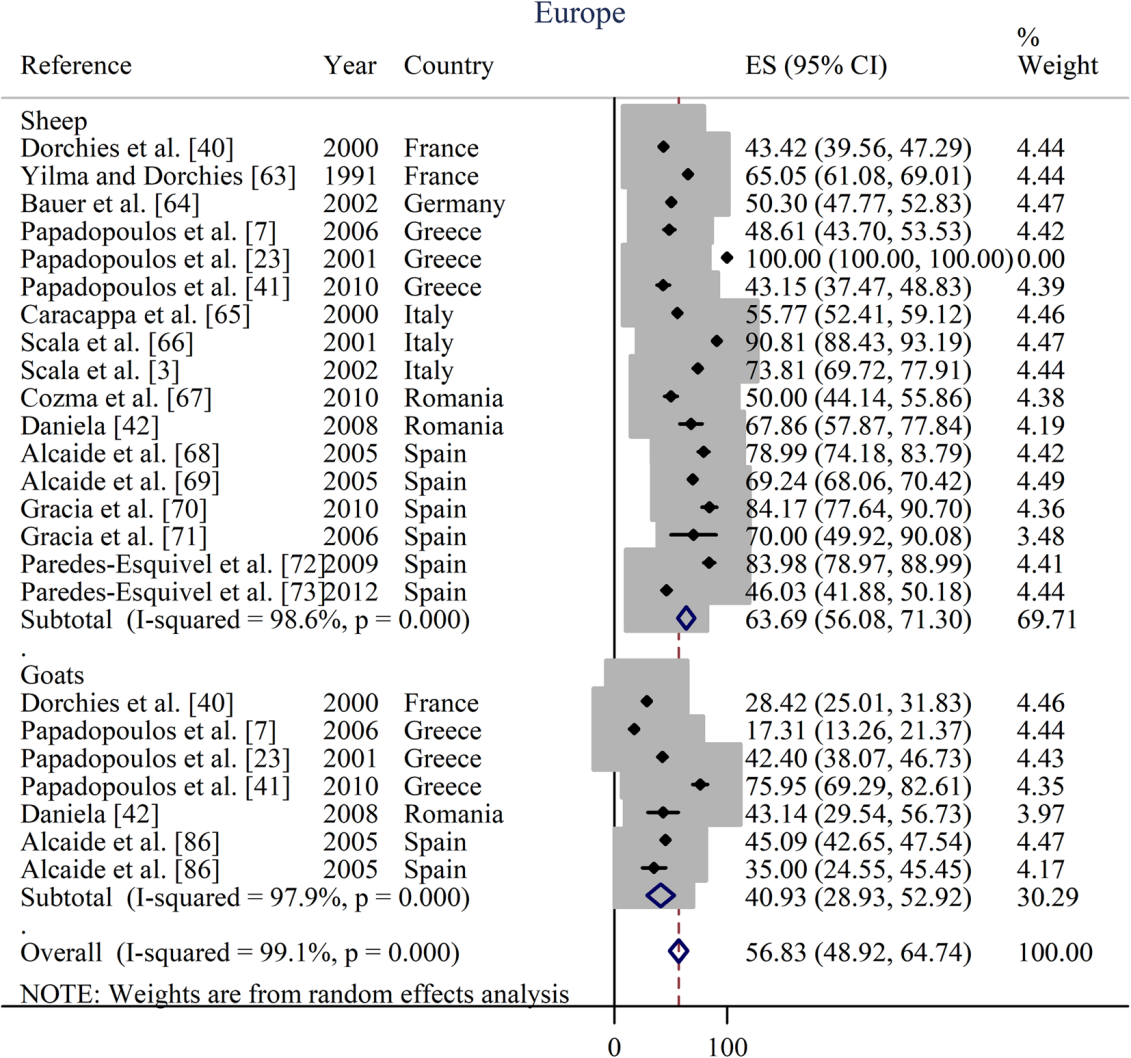
Forest plot of the prevalence estimates of oestrosis caused by *Oestrus ovis* in sheep and goats amongst studies conducted in Europe

**Fig. 6:**
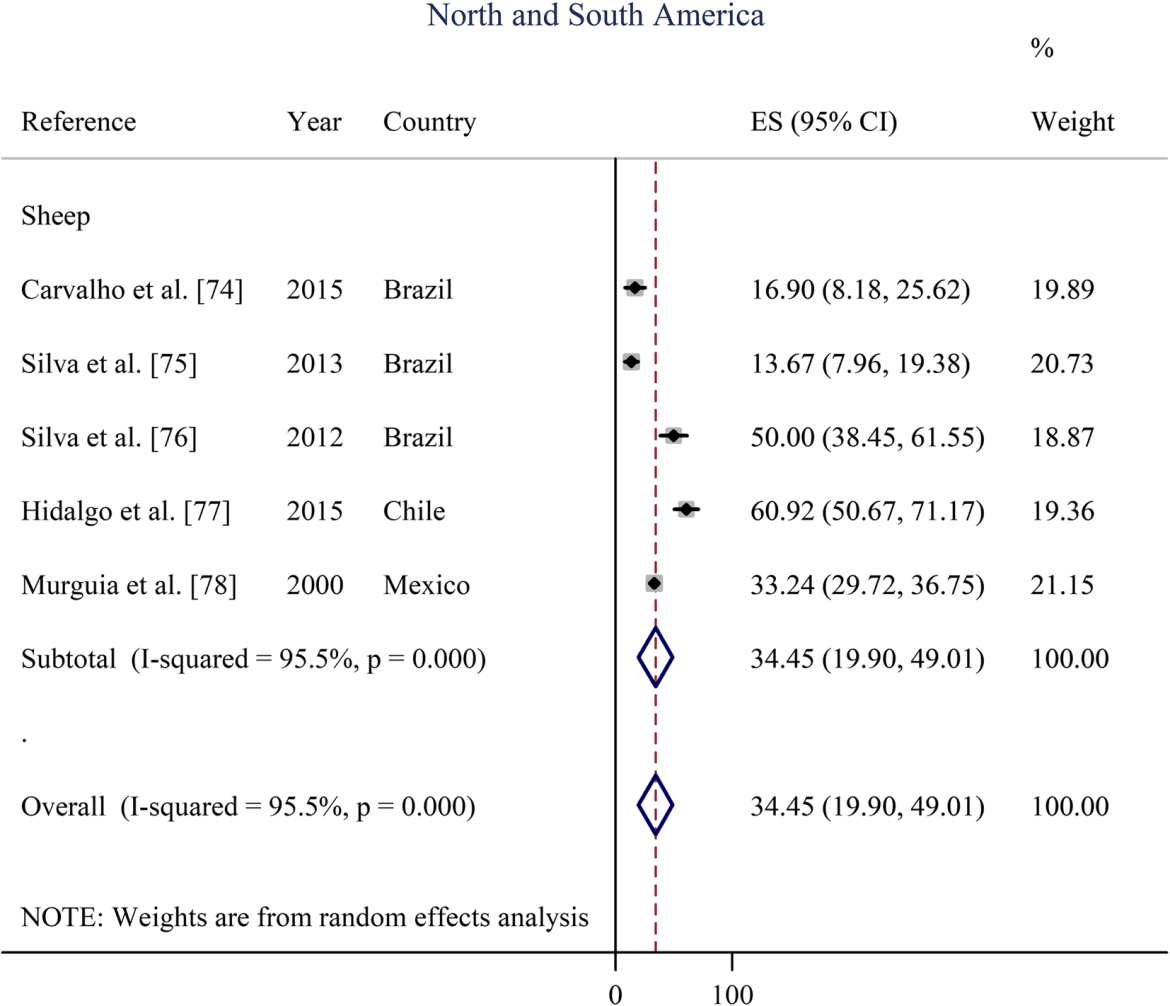
Forest plot of the prevalence estimates of oestrosis caused by *Oestrus ovis* in sheep amongst studies conducted in North and South America. Note that there are no data on oestrus in goats between 1970 and 2018 in both North and South America

Prevalence estimates from individual contributing studies according to world region are outlined in Figs. 3, 4, 5, 6 and Table 2. The lowest individual prevalence of oestrosis in sheep was reported as 5.88% (95% CI: 3.91–7.86%) in Saudi Arabia [19] and the highest individual prevalence was reported as 100% (95% CI: 100–100%) in Greece [23]. In goats, the lowest individual prevalence was reported as 13.06% (95% CI: 11.59–14.54%) in Iran [84] and the highest individual prevalence was reported as 88.40% (95% CI: 85.38–91.42%) in Ethiopia [30]. The longest study duration was 80 months [74] while the shortest study duration was 2 months [39].

Age and sex-related prevalence are summarised in Table 5. For determining the age effect, two age groups were selected. The estimated pooled prevalence of oestrosis was 49.53% (95% CI: 38.02–61.03%) in adult animals and 38.30% (95% CI: 27.21–49.38%) in young animals. The overall estimated pooled prevalence of oestrosis was 48.74% (95% CI: 31.16–66.33%) in female animals and 39.55% (95% CI: 27.73–51.36%) in male animals.

Pooled prevalence based on the origin of samples, methods of detection and study duration are shown in Table 5. The overall estimated pooled prevalence of oestrosis in sheep and goats slaughtered in abattoirs was 47.72% (95% CI: 40.53–44.90%) while the prevalence in farmed animals was 50.78% (95% CI: 36.49–65.07%). Prevalence was 47.16% (95% CI: 39.81–54.51%), 50.87% (95% CI: 39.09–62.65%) and 56.18% (95% CI: 37.80–74.57%) by necropsy, serology and others methods of diagnosis, respectively, to detect oestrosis in sheep and goats. Studies conducted for > 6 to ≤ 12 months had higher prevalence 52.23% (95% CI: 40.57–63.89%) than the study conducted for ≤ 6 months [49.19% (95% CI: 37.73–60.64%)] and > 12 months [41.78% (95% CI: 30.37–53.19%)].

### Effect of ambient temperature

**Fig. 7:**
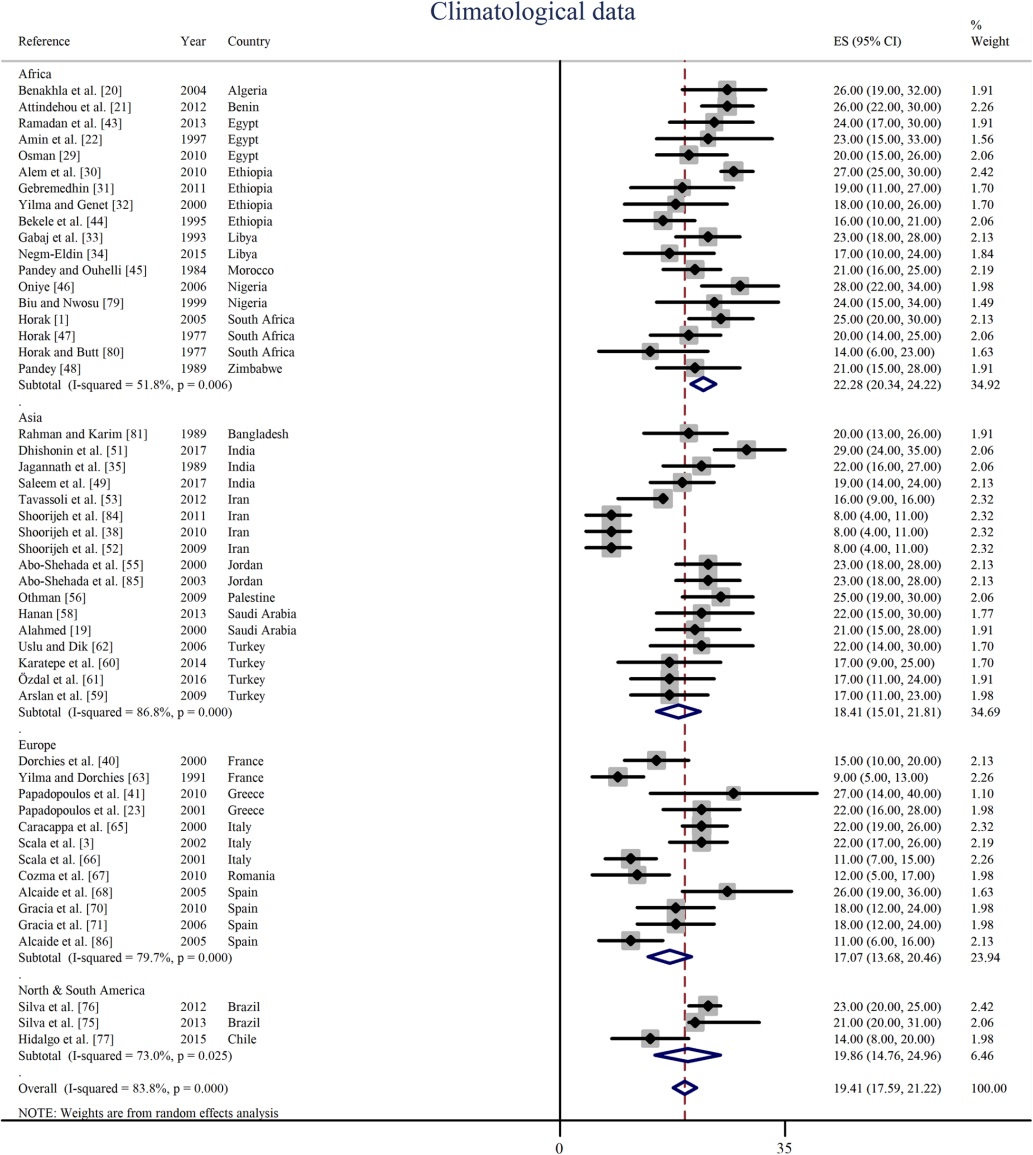
Ambient environmental temperature at which the peak prevalence of oestrosis found in sheep and goats at different continents and countries of the world

Peak prevalence season of oestrosis in sheep and goats and ambient environmental temperature during that particular period of the year was obtained from 50 articles representing 23 countries of the world. The average ambient temperature during the peak infestation period was 22 °C (95% CI: 20–24 °C) in Africa, 18 °C (95% CI: 15–22 °C) in Asia, 17 °C (95% CI: 14–20 °C) in Europe and 20 °C (95% CI: 15–25 °C) in South America. Overall, the random effect estimated pooled global ambient temperature was 19 °C (95% CI: 18–21 °C) with significant heterogeneity (*I*^2^= 83.8%, *P* < 0.0001). The lowest temperature at which the peak prevalence of oestrosis observed was 10 °C (95% CI: 6–14 °C) in Iran while the highest temperature at which the peak prevalence found was 27 °C (95% CI: 22–32 °C) in Nigeria. The ambient temperature at which the peak prevalence was observed are shown in Fig. 7 and country-wise results in Table 6. Results of sub-group analysis of peak prevalence estimates based on ambient temperature are shown in Additional file 5: Figure S2.

**Table 6:** Estimated pooled temperature at which the peak prevalence of oestrosis reported in sheep and goats in different world regions

Country	T (°C)	95% CI	Heterogeneity (*χ*^2^)	*I*^2^ (%)	*P*-value
Algeria	26	19–32	0	–	–
Bangladesh	20	13–26	0	–	–
Benin	26	22–30	0	–	–
Brazil	22	20–25	0.42	0.0	0.516
Chile	14	7–20	0	–	–
Egypt	22	18–26	0.92	0.0	0.632
Ethiopia	20	14–27	17.29	82.6	0.001
France	12	6–18	3.37	70.4	0.066
Greece	23	17–28	0.47	0.0	0.494
India	23	17–29	7.16	72.1	0.028
Iran	10	6–14	15.05	80.1	0.002
Italy	18	11–26	19.67	89.8	< 0.0001
Jordan	23	19–27	0	0.0	1.000
Libya	20	15–26	1.87	46.5	0.172
Morocco	21	16–25	0	–	–
Nigeria	27	22–32	0.49	0.0	0.485
Palestine	25	19–30	0	–	–
Romania	12	6–18	0	–	–
Saudi Arabia	22	17–27	0.04	0.0	0.843
South Africa	20	14–26	5.15	61.2	0.076
Spain	18	12–23	9.93	69.8	0.019
Turkey	18	15–21	1.22	0.0	0.749
Zimbabwe	21	15–28	0	–	–

*Abbreviations*: CI, confidence interval; *χ*^2^, Cochran’s *Q* Chi square; *I*^2^, inverse variance index; –, no interaction due to having single study; T, pooled average temperature

### Source of heterogeneity

Six sources of heterogeneity in prevalence of oestrosis in sheep and goats were observed. These were: world region (*P* < 0.0001); age (*P* < 0.0001); sex (*P* < 0.0001); origin of the sample (*P* < 0.0001); method of detection (*P* < 0.0001); and study duration (*P* < 0.0001) (Table 5).

**Fig. 8:**
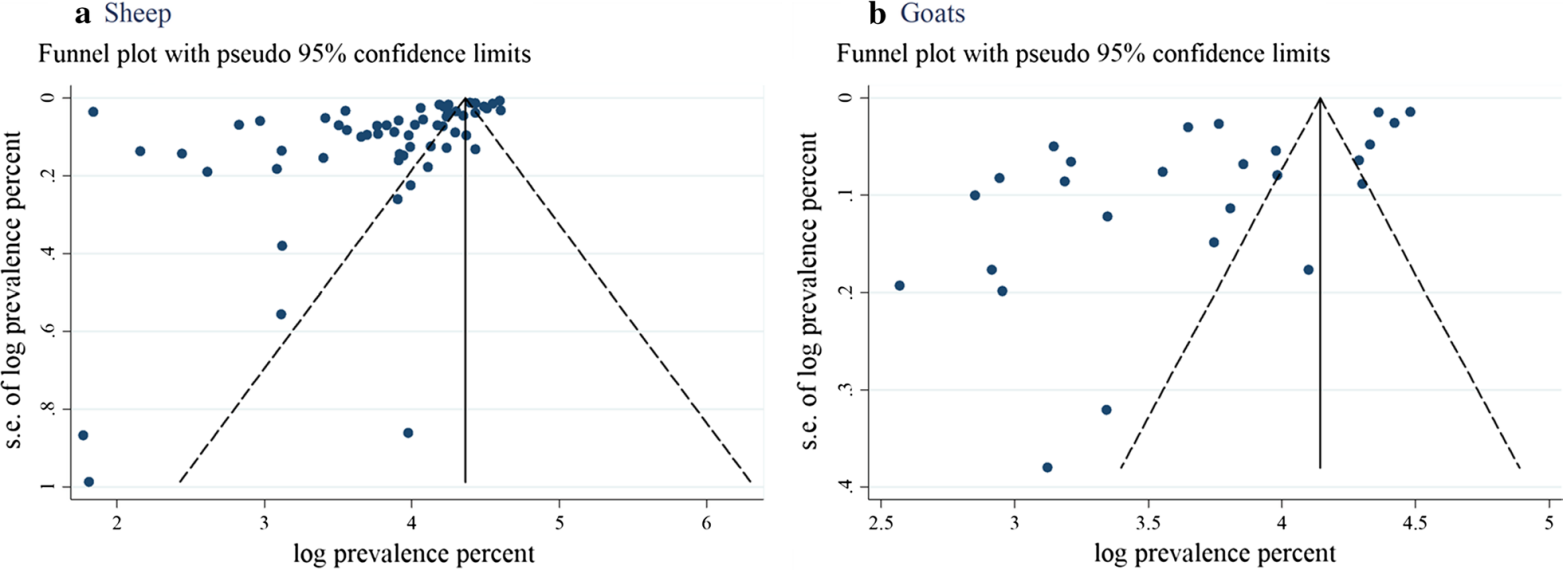
Funnel plot for examination of publication bias of the prevalence estimates of oestrosis in sheep (**a**) and goat (**b**). *Abbreviation*: s.e., standard error

Overall, there was a high level of heterogeneity in most pooled prevalence estimates (*I*^2^ > 80%). The expanse of publication bias in the selected studies was measured separately for sheep (Fig. 8a) and goats (Fig. 8b). Both funnel plots appeared with the asymmetrical appearance with a gap in the right bottom side of the graph and many points fall outside of the funnels in the left side, indicating publication bias. The estimated bias co-efficient in sheep was 4.56 (95% CI: 4.45–4.67) with a standard error 0.055 providing a *P*-value of < 0.0001 while the estimated bias coefficient in goats was 4.34 (95% CI: 4.07–4.61) with a standard error 0.129 and a *P*-value of < 0.0001. Bias assessment checklist and scores of individual studies are shown in Additional file 2: Table S2.

## Discussion

This study summarises the prevalence of oestrosis in sheep and goats in global and regional levels based on a large population (*n* = 59,086; sheep: 40,870 and goats: 18,216) derived from 26 countries of five continents of the world that enabled the assessment of reliable prevalence estimates according to the study objectives. To the best of author’s knowledge, this is the first meta-analysis of the global prevalence of oestrosis in sheep and goats.

The global prevalence of oestrosis in sheep and goats is high with an estimated pooled prevalence of 48.25% (95% CI: 41.82–54.67%) across 66 published reports. Around the globe, Europe had the highest disease burden 56.83% (95% CI: 48.92–64.74%) and North and South America had the lowest disease burden at 34.46% (95% CI: 19.90–49.01%). Mexico was the only country of North America that reported oestrosis in sheep; therefore, data for Mexico were compiled with the data from South American countries to estimate the pooled prevalence for meaningful analysis. There could be several reasons for the high prevalence of oestrosis in sheep and goats such as a pasture-based farming system, the presence of favourable climatic conditions for flies and a limited level of flock/herd monitoring as farmers usually provide less attention to individual sheep and goats as they do for other farm animals. Moreover, many studies were conducted in slaughterhouses in different countries which quickly revealed the infestation of *O. ovis* larvae from the nasal sinuses of sheep and goats [20–22].

The reported ambient environmental temperature at which the peak prevalence of oestrosis observed in sheep and goats varied dramatically across regions and countries [51, 52]. This variation could be due to a variable life-cycle of *O. ovis*, which may differ from a couple of weeks to several months in different geographical regions based on climatic and environmental conditions. An earlier study reported that flies became active when the environmental temperatures were above 12–18 °C and larvae showed quick movement and dynamic foraging behaviour at 19–22 °C [87]; this is in agreement with the finding of this meta-analysis. On the other hand, Cepeda-Palacios et al. [87] also reported that larvae underwent hypobiosis when the temperature was around 5 °C, and in this meta-analysis, no peak prevalence was observed under a temperature of 8 °C [38, 52]. However, it is known that *O. ovis* larvae are capable of adjusting their biology according to ambient environmental conditions and that range is quite large [88].

The estimated pooled prevalence indicates that oestrosis is more prevalent in sheep than in goats. These results are in agreement with other studies that reported a higher prevalence of oestrosis in sheep than in goats [40, 42, 51]. The higher prevalence in sheep could be due to more host specificity of *O. ovis* to sheep. Another speculation could be the moistness of muzzle. It is known that goats consume less water than sheep; therefore, their noses are usually less humid than in sheep. This higher humidity may help the larvae to survive more easily in sheep [89]. Additionally, sheep and goats also differ in respect to immune response and may have different responsiveness to an adult fly strike. The host-related odour difference between sheep and goats may also play a significant role in oestrosis prevalence [90].

The estimated pooled prevalence of oestrosis was higher in adults than in young animals. This finding agrees with the results of many studies [21, 29, 34, 54, 79], but not with several others [30, 59, 60]. A possible reason for the high prevalence in adult animals may be that adult animals are more attractive to the female flies and the surface area in the nasal orifice of adult animals are much broader than of young animals. Furthermore, the respiration rates of adult animals are slower than of young that may offer assistance the female fly to oviposit and to larvae to crawl into the nasal sinuses. Moreover, a young animal may have maternally derived antibodies against oestrosis [91]. Conversely, several reports observed that age of the animal (> 13 weeks) positively influenced the immune response (humoral or cellular) development against *O. ovis* in sheep, which tends to vary with infestation load and other factors [92–94]. Likewise, another study reported that lamb could have a higher infestation and larval burden and are significantly responsible for maintaining oestrosis due to less developed immune competency [95].

Oestrosis was higher in female animals than in male animals based on estimated pooled prevalence. This is plausibly due to an increased density of females to males in flocks or to the physiological differences between males and females or a particular habit of female animals which facilitates their infestation by *O. ovis* larvae [34]. Conversely, the effect of sex on the prevalence of oestrosis was not significant in one study [31], and a higher prevalence in males was observed in another study [34].

The difference in prevalence considering the origin of the sample, the method of detection and the duration of the study was also significant. Prevalence was higher in farm-based studies compared to abattoirs; this may be due to the method of detection, as farm-based studies mostly rely upon serology [7] and there is a high chance that the fly can attack many animals of a flock in a particular farm in an endemic area. On the other hand, animals slaughtered in an abattoir usually come from different regions and different farms; therefore, the pooled prevalence can be lower. However, year-round surveillance can give an actual prevalence estimate.

Funnel plot asymmetry reveals strong evidence of the presence of publications bias. However, there are many other reasons for funnel plot asymmetry like true heterogeneity, location, data irregularity and artefacts, or even by chance [28].

### Limitations

This study has several limitations. First, no report on oestrosis of sheep and goats was found in the continent of Australia within the range of this meta-analysis. Thus a reflection of the prevalence from these regions could not be obtained. Moreover, only four studies from South America and one study from North America were obtained, and all five articles reported the prevalence of sheep oestrosis, so the prevalence of goat oestrosis could not be estimated. Secondly, most of the studies were conducted in head samples obtained directly from abattoirs, so in some cases it could be difficult to determine the actual age and sex of the animals and exact prevalence estimation. Thirdly, non-English articles, unpublished articles, case reports and results of experimental trials were not included in this meta-analysis. Fourthly, due to unavailability of data regarding age, sex and peak prevalence season in every article, all articles could not be covered for pooled prevalence estimations. Finally, the data displayed a significant heterogeneity between studies even within a particular region.

## Conclusions

Results of the global meta-analysis show a very high burden of oestrosis reported in many regions, especially in northern Africa and southern Europe. The results also indicate that the disease is more prevalent in sheep than in goats. The main implication of these results is that screening tests for *O. ovis* and treatment should be routinely carried out in sheep and goat flocks in high disease burden regions to improve animal productivity and minimise the potential zoonotic risk to humans. Measures should also be implemented to take adequate preventive measures against *O. ovis* infestation. As oestrosis is found more prevalently in adult animals, vaccine development and immunisation at the young stage of life may prevent the disease. Moreover, reports on the prevalence of oestrosis in sheep and goats are still not available from many regions; therefore, epidemiological surveillance is needed for estimating the disease burden and for controlling the disease. Additionally, factors that contribute to the prevalence estimate should be handled appropriately in any survey to estimate the true prevalence of oestrosis.

## Additional files

**Additional file 1: Table S1.** PRISMA 2009 checklist.

**Additional file 2: Text S1.** Quality assessment checklist of individual studies. **Table S2.** Quality score of studies included in the meta-analysis. **Figure S1.** Frequency distribution of eligible studies characteristics.

**Additional file 3: Text S2.** List of the articles excluded in the present meta-analysis with justification.

**Additional file 4: Text S3.** List of the articles included in the present meta-analysis.

**Additional file 5: Figure S2.** Sub-group analysis of ambient environmental temperature at which the peak prevalence of oestrosis was found in sheep and goats in different continents and countries of the world.

## Data Availability

Important datasets that support the conclusions of this article are included within the article and in additional files.
