# Supplementary material for: The global and regional prevalence of oestrosis in sheep and goats: a systematic review of articles and meta-analysis
Source: Parasit Vectors. 2019 Jul 12;12:346. doi: 10.1186/s13071-019-3597-2 (PMC6625052; doi:10.1186/s13071-019-3597-2)
Supplement: Supplementary file 2 — Additional file 2: Text S1. Quality assessment checklist of individual studies. Table S2. Quality score of studies included in the meta-analysis. Figure S1. Frequency distribution of eligible studies characteristics. [file 13071_2019_3597_MOESM2_ESM.docx]

**Additional file 2: Text S1.** Quality assessment checklist

The following items were examined and given a score based on a simple scale system (1 for ''yes'', 0 for ''no'').

1. Was the research objective clearly stated?
2. Was the sampling area clearly described with reference to the location?
3. Was the period of the study stated?
4. Was the target sample a close representation of the general population?
5. Was some form of random selection used to select the samples
6. Was a minimum sample size calculated?
7. Were the sample processing and diagnostic method clearly described?
8. Were the subjects categorised by sex?
9. Were the subjects categorised by age and were the age categories clearly defined?
10. Were the subjects represent peak prevalent season?

The quality index score for each study was calculated by dividing the study quality score by 10.

**Additional file 2: Table S2.** Quality score of studies included in the meta-analysis

| Author | Year | Country | Quality score | Quality index score |
| --- | --- | --- | --- | --- |
| Benakhla et al. [20] | 2004 | Algeria | 7 | 0.7 |
| Attindehou et al. [21] | 2012 | Benin | 7 | 0.7 |
| Amin et al. [22] | 1997 | Egypt | 4 | 0.4 |
| Osman [29] | 2010 | Egypt | 5 | 0.5 |
| Ramadan et al. [43] | 2013 | Egypt | 6 | 0.6 |
| Alem et al. [30] | 2010 | Ethiopia | 7 | 0.7 |
| Bekele et al. [44] | 1995 | Ethiopia | 5 | 0.5 |
| Gebremedhin [31] | 2011 | Ethiopia | 8 | 0.8 |
| Yilma and Genet [32] | 2000 | Ethiopia | 8 | 0.8 |
| Gabaj et al. [33] | 1993 | Libya | 4 | 0.4 |
| Negm-Eldin [34] | 2015 | Libya | 7 | 0.7 |
| Pandey and Ouhelli [45] | 1984 | Morocco | 5 | 0.5 |
| Oniye [46] | 2006 | Nigeria | 7 | 0.7 |
| Horak [47] | 1977 | South Africa | 5 | 0.5 |
| Horak [1] | 2005 | South Africa | 6 | 0.6 |
| Pandey [48] | 1989 | Zimbabwe | 7 | 0.7 |
| Biu and Nwosu [79] | 1999 | Nigeria | 5 | 0.5 |
| Horak and Butt [80] | 1977 | South Africa | 4 | 0.4 |
| Saleem et al. [49] | 2017 | India | 5 | 0.5 |
| Sharma et al. [50] | 2012 | India | 1 | 0.1 |
| Dhishonin et al. [51] | 2017 | India | 4 | 0.4 |
| Jagannath et al. [35] | 1989 | India | 4 | 0.4 |
| Pathak [36] | 1992 | India | 3 | 0.3 |
| Dehghani et al. [37] | 2012 | Iran | 6 | 0.6 |
| Shoorijeh et al. [38] | 2010 | Iran | 8 | 0.8 |
| Shoorijeh et al. [52] | 2009 | Iran | 8 | 0.8 |
| Tavassoli et al. [53] | 2012 | Iran | 5 | 0.5 |
| AL-Ubeidi et al. [54] | 2017 | Iraq | 4 | 0.4 |
| Abo-Shehada et al. [55] | 2000 | Jordan | 6 | 0.6 |
| Othman [56] | 2009 | Palestine | 5 | 0.5 |
| Alahmed [19] | 2000 | Saudi Arabia | 4 | 0.4 |
| Alikhan et al. [57] | 2018 | Saudi Arabia | 3 | 0.3 |
| Hanan [58] | 2013 | Saudi Arabia | 7 | 0.7 |
| Arslan et al. [59] | 2009 | Turkey | 9 | 0.9 |
| Ipek and Altan [39] | 2017 | Turkey | 5 | 0.5 |
| Karatepe et al. [60] | 2014 | Turkey | 7 | 0.7 |
| Özdal et al. [61] | 2016 | Turkey | 9 | 0.9 |
| Uslu and Dik [62] | 2006 | Turkey | 9 | 0.9 |
| Rahman and Karim [81] | 1989 | Bangladesh | 5 | 0.5 |
| Huq [82] | 1983 | Bangladesh | 3 | 0.3 |
| Jumde and Dixit [83] | 2012 | India | 2 | 0.2 |
| Shoorijeh et al. [84] | 2011 | Iran | 7 | 0.7 |
| Abo-Shehada et al. [85] | 2003 | Jordan | 6 | 0.6 |
| Dorchies et al. [40] | 2000 | France | 7 | 0.7 |
| Yilma and Dorchies [63] | 1991 | France | 7 | 0.7 |
| Bauer et al. [64] | 2002 | Germany | 5 | 0.5 |
| Papadopoulos et al. [7] | 2006 | Greece | 6 | 0.6 |
| Papadopoulos et al. [23] | 2001 | Greece | 7 | 0.7 |
| Papadopoulos et al. [41] | 2010 | Greece | 7 | 0.7 |
| Caracappa et al. [65] | 2000 | Italy | 5 | 0.5 |
| Scala et al. [66] | 2001 | Italy | 6 | 0.6 |
| Scala et al. [3] | 2002 | Italy | 5 | 0.5 |
| Cozma et al. [67] | 2010 | Romania | 4 | 0.4 |
| Daniela [42] | 2008 | Romania | 2 | 0.2 |
| Alcaide et al. [68] | 2005 | Spain | 8 | 0.8 |
| Alcaide et al. [69] | 2005 | Spain | 5 | 0.5 |
| Gracia et al. [70] | 2010 | Spain | 6 | 0.6 |
| Gracia et al. [71] | 2006 | Spain | 3 | 0.3 |
| Paredes-Esquivel et al. [72] | 2009 | Spain | 4 | 0.4 |
| Paredes-Esquivel et al. [73] | 2012 | Spain | 5 | 0.5 |
| Alcaide et al. [86] | 2005 | Spain | 8 | 0.8 |
| Carvalho et al. [74] | 2015 | Brazil | 4 | 0.4 |
| Silva et al. [75] | 2013 | Brazil | 5 | 0.5 |
| Silva et al. [76] | 2012 | Brazil | 5 | 0.5 |
| Hidalgo et al. [77] | 2015 | Chile | 9 | 0.9 |
| Murguía et al. [78] | 2000 | Mexico | 7 | 0.7 |

**Additional file 2: Figure S1.** Frequency distribution of eligible studies characteristics
