# Supplementary material for: The global and regional prevalence of oestrosis in sheep and goats: a systematic review of articles and meta-analysis
Source: Parasit Vectors. 2019 Jul 12;12:346. doi: 10.1186/s13071-019-3597-2 (PMC6625052; doi:10.1186/s13071-019-3597-2)
Supplement: Supplementary file 3 — Additional file 3: Text S2. List of the articles excluded in the present meta-analysis with justification. [file 13071_2019_3597_MOESM3_ESM.docx]

**Additional file 3: Text S2** Full text articles excluded

**Case report: (n=7)**

1. Gunalan S, Kamaliah G, Wan S, Rozita A, Rugayah M, Osman M, et al. Sheep oestrosis (Oestrus Ovis, Diptera: Oestridae) in Damara crossbred sheep. Malays J Vet Res. 2011;2:41-9.

2. Gomez-Puerta LA, Alroy KA, Ticona DS, Lopez-Urbina MT, Gonzalez AE. A case of nasal myiasis due to Oestrus ovis (Diptera: Oestridae) in a llama (Lama glama). Revista Brasileira de Parasitologia Veterinária. 2013;22 4:608-10.

3. Godara R, Sharma R, Sharma C. Aberrant infestation of goat mandibles with Oestrus ovis larvae. Tropical animal health and production. 2010;42 1:137.

4. Sharma N, Nayakwadi S, Pawaiya RS, Kumar S, Tailie WA, Paul S, et al. Parasitic encephalomyelitis in goats due to aberrant infestation with botfly Oestrous ovis larvae. Adv Anim Vet Sci. 2014;2 35:8-11.

5. Madhu D, Sudhakar N, Maurya P, Manjunathachar H, Sahu S, Pawde A. Nasal Oestrosis in a Jamunapari goat. Journal of parasitic diseases. 2014;38 4:396-8.

6. Hasheminasab SS, Talvar HM, Wright I. First case report of Oestrus ovis in horns of goat despite the healthy sinuses and nasal passages with its morphological study from Iran. Indian Journal of Animal Research. 2016;50 2:278-80.

7. Allaie I, Wani Z, Malik A, Shahardar R, Zulhuma M. Oestrus ovis larvae in nasal cavity of sheep: a case report. Journal of parasitic diseases. 2016;40 4:1221-2.

**Individual prevalence data is not available: (n=7)**

8. Mozaffari AA, Shojaeepour S, Ghahremani Ghareh Cheshmeh S. High mortality rate due to false gid in a sheep herd. ISRN veterinary science. 2013;2013.

9. Papadopoulos E, Dvořák L. Oestrus ovis (linnaeus, 1761)(diptera, oestridae), first record of an adult from Greece. Parnassiana Archives. 2015;3 1:3-6.

10. Fonseca O, Moya VM, de las Nieves Montano D, Centelles Y, Percedo MI, Alfonso P. Spatial modeling of oestrosis in sheep in Guantánamo province, Cuba. Small Ruminant Research. 2018;164:32-8.

11. Gaaboub IA. The distribution and seasonal dynamics of Oestrus ovis Linne infesting the nasal cavities and sinuses of sheep in Egypt. Veterinary Parasitology. 1978;4 1:79-82.

12. Jagannath M, Cozab N, Rahman SA, Honnappa T. Serodiagnosis of oestrus-ovis infestation in sheep and goats. Indian Journal of Animal Sciences. 1989;59 10:1220-4.

13. Mboera L, Kitalyi J: Diseases of small ruminants in central Tanzania. In: *Small Ruminant Research and Development in Africa: Proceedings of the Second Biennial Conference of the African Small Ruminant Research Network: AICC, Arusha, Tanzania, 7-11 December 19921994*: ILRI (aka ILCA and ILRAD): 117.

14. Kettle P. A study on the sheep botfly, Oestrus ovis (Diptera: Oestridae) in New Zealand. New Zealand Entomologist. 1973;5 2:185-91.

**Experimental trial: (n=8)**

15. Dorchies P, Wahetra S, Lepetitcolin E, Prevot F, Grisez C, Bergeaud J, et al. The relationship between nasal myiasis and the prevalence of enzootic nasal tumours and the effects of treatment of Oestrus ovis and milk production in dairy ewes of Roquefort cheese area. Veterinary parasitology. 2003;113 2:169-74.

16. Jacquiet P, Dorchies P. Towards a lower prevalence of Oestrus ovis infections in sheep in a temperate climate (south west France). Veterinary Research. 2002;33 5:449-53.

17. Suarez VH, Busetti MR, Miranda AO, Prévot F, Jacquiet P. Epidemiology of Oestrus ovis infection of sheep in Argentina's Western Pampas. Parasite. 2004;11 4:405-10.

18. Cepeda-Palacios R, Avila A, Ramırez-Orduna R, Dorchies P. Estimation of the growth patterns of Oestrus ovis L. larvae hosted by goats in Baja California Sur, Mexico. Veterinary parasitology. 1999;86 2:119-26.

19. Cepeda-Palacios R, Servín R, Ramírez-Orduña J, Ascencio F, Dorchies P, Angulo-Valadez C. In vitro and in vivo effects of neem tree (Azadirachta indica A. Juss) products on larvae of the sheep nose bot fly (Oestrus ovis L. Díptera: Oestridae). Veterinary parasitology. 2014;200 1-2:225-8.

20. Hoste H, Lemercier P, Jacquiet P, Dorchies P, Lespine A, Alvinerie M. Efficacy of eprinomectin pour-on against gastrointestinal nematodes and the nasal bot fly (Oestrus ovis) in sheep. Veterinary record. 2004;154 25:782-5.

21. Lucientes J, Castillo JA, Ferrer LM, Peribáñez MA, Ferrer-Dufol M, Gracia-Salinas MaJ. Efficacy of orally administered ivermectin against larval stages of Oestrus ovis in sheep. Veterinary parasitology. 1998;75 2-3:255-9.

22. Habela M, Moreno A, Gragera-Slikker A, Gomez J, Montes G, Rodriguez P, et al. Efficacy of eprinomectin pour-on in naturally Oestrus ovis infested merino sheep in Extremadura, South-West Spain. Parasitology research. 2006;99 3:275-80.

**Others: (n=4)**

23. Moreno V, Pérez JM, Moreno PA, Granados JE, Ruiz-Martinez I, Soriguer RC, et al. Oestrid myiasis in European mouflon from Spain. Journal of wildlife diseases. 1999;35 1:78-81.

24. Howard G. Prevalence of nasal bots (Diptera: Oestridae) in some Zambian Hartebeest. Journal of wildlife diseases. 1977;13 4:400-4.

25. Barroso P, Ruiz-de-Ybáñez R, Martínez-Carrasco C, Gens MJ, Escribano F, Sánchez A, et al. First report of oestrosis in aoudad from southeastern Spain. Parasitology research. 2017;116 7:2053-5.

26. Dehghani R, Sedaghat MM, Esmaeli N, Ghasemi A. Myiasis among slaughtered animals in Kashan, Iran: descriptive a veterinary entomological problem in the tropics. Iranian Journal of Veterinary Science and Technology. 2014;4 1:19-28.

**Non-English (n=1)**

27. Belem A, Rouille D. Oestrus ovis infestation of sheep and goats in Burkina faso. Revue d’Elevage et de Medecine Veterinaire. 1988;139:701-4.
