# Supplementary material for: The global and regional prevalence of oestrosis in sheep and goats: a systematic review of articles and meta-analysis
Source: Parasit Vectors. 2019 Jul 12;12:346. doi: 10.1186/s13071-019-3597-2 (PMC6625052; doi:10.1186/s13071-019-3597-2)
Supplement: Supplementary file 4 — Additional file 4: Text S3. List of the articles included in the present meta-analysis. [file 13071_2019_3597_MOESM4_ESM.docx]

**Additional file 4: Text S3.** Articles included

1. Horak IG. Parasites of domestic and wild animals in South Africa. I. Oestrus ovis in sheep. Onderstepoort Journal of Veterinary Research. 1977; 44 2:55-64.
2. Papadopoulos E, Prevot F, Diakou A, Dorchies P. Comparison of infection rates of Oestrus ovis between sheep and goats kept in mixed flocks. Veterinary parasitology. 2006;138 3-4:382-5.
3. Alahmed A. Seasonal infestation of Oestrus ovis larvae in sheep heads in central region of Saudi Arabia. . Journal of the Egyptian Society of Parasitology. 2000;30 3:895-901.
4. Papadopoulos E, Prevot F, Jacquiet P, Duranton C, Bergeaud J, Kalaitzakis E, et al. Seasonal variation of Oestrus ovis-specific antibodies in sheep and goats mixed flocks in Greece. Veterinary parasitology. 2001;95 1:73-7.
5. Shoorijeh JS, Tamadon A, Negahban S, Behzadi M. Prevalence of Oestrus ovis in goats of Shiraz, southern Iran. Veterinarski arhiv. 2011;81 1:43-9.
6. Alem F, Kumsa B, Degefu H. Oestrus ovis larval myiasis among sheep and goats in Central Oromia, Ethiopia. Tropical animal health and production. 2010;42 4:697-703.
7. Carvalho RS, Ruivo MA, Colli MHA, Pereira V, Martinez AC, Mazzucatto BC, et al. Occurrences of Oestrus ovis parasitism in necropsied sheep in the Umuarama microregion, Paraná, Brazil. Revista Brasileira de Parasitologia Veterinária. 2015;24 3:370-4.
8. İpek DNS, Altan S. Use of semi-nested PCR and rhinoscopy for the diagnosis of oestrosis. Small ruminant research. 2017;150:76-9.
9. Benakhla A, Sedraoui S, Benouareth D, Cabaret J, Boulard C. Epidemiology of sheep infection by Oestrus ovis in Algeria. Parasite. 2004;11 2:235-8.
10. Attindehou S, Salifou S, Gbangboche A, Abiola F. Prevalence of the small ruminant’s oestrosis in Benin. Journal of Animal and Veterinary Advances. 2012.
11. Amin A, Morsy T, Shoukry A, Mazyad S. Oestrid head maggots in slaughtered sheep in Cairo abattoir. Journal of the Egyptian Society of Parasitology. 1997;27 3:855-61.
12. Shoorijeh SJ, Negahban S, Tamadon A, Behzadi MA. Prevalence and intensity of Oestrus ovis in sheep of Shiraz, southern Iran. Tropical animal health and production. 2009;41 7:1259.
13. Dhishonin S, Babu RN, Ramani R, Porteen K, Rao VA, Abraham RJ, et al. A Survey of Disease Conditions in Sheep and Goats Slaughtered at Coimbatore District Slaughter House, Tamil Nadu, India. Int J Curr Microbiol App Sci. 2017;6 10:3692-9.
14. Shoorijeh JS, Tamadon A, Negahban S, Behzadi M, Biglari S. Seasonal infection rates of Oestrus ovis. Online Journal of Veterinary Research. 2010;14 302-310.
15. Dorchies P, Bergeaud J, Tabouret G, Duranton C, Prevot F, Jacquiet P. Prevalence and larval burden of Oestrus ovis (Linne 1761) in sheep and goats in northern Mediterranean region of France. Veterinary Parasitology. 2000;88 3-4:269-73.
16. Daniela M. The prevalence of wormy sinusitis in goats from the west side of our country. Scientific Papers Animal Science and Biotechnologies. 2008;41 2:762-4.
17. AL-Ubeidi N, ALani AJ, Al-kennany E. Detection of nasal bot fly larvae in slaughtered sheep of Ninevah governorate, Iraq. Basrah Journal of Veterinary Research. 2017;16 2:240-7.
18. Biu AA, Nwosu CO. Incidence of Oestrus ovis infestation in Borno-White Sahel goats in the semi-arid zone of Nigeria. Veterinary research. 1999;30 1:109-12.
19. Negm-Eldin MM, Elmadawy RS, Hanan GM. Oestrus ovis larval infestation among sheep and goats of Green Mountain areas in Libya. Journal of Advanced Veterinary and Animal Research. 2015;2 4:382-7.
20. Osman M: Epidemiological studies on Oestrus ovis infection, in sheep and goats in Shalatin area, Red Sea Governorate, Egypt. In: *Proceedings of the 3rd Scientific Conference of Animal Wealth Research in the Middle East and North Africa, Foreign Agricultural Relations (FAR), Egypt, 29 November-1 December 20102010*: Massive Conferences and Trade Fairs: 25-40.
21. Karatepe B, Karatepe M, Güler S. Epidemiology of Oestrus ovis L. infestation in sheep in Nigde province, Turkey. Revue Med Vet. 2014;165 7:225-30.
22. Arslan M, Kara M, Gicik Y. Epidemiology of Oestrus ovis infestations in sheep in Kars province of north-eastern Turkey. Tropical animal health and production. 2009;41 3:299.
23. Silva BFd, Bassetto CC, Amarante AFTd. Immune humoral response of young lambs naturally infested by Oestrus ovis (Diptera: Oestridae). Revista Brasileira de Parasitologia Veterinária. 2018; ahead.
24. Gebremedhin EZ. Prevalence of ovine and caprine oestrosis in Ambo, Ethiopia. Tropical animal health and production. 2011;43 1:265-70.
25. Ramadan MY, Khater HF, Omer SF, Rahman AA: Epidemiology of Oestrus ovis Infesting Egyptian Sheep. In: *XX International Congress of Mediterranean Federation of Health and Production of Ruminants2013*.
26. Bekele T, Mukasa-Mugerwa E. Oestrus ovis infection in Ethiopian highland sheep. Veterinary research communications. 1994;18 6:439-42.
27. Yilma J, Genet A. Epidemiology of the sheep nasal bot, Oestrus ovis (Diptera: Oestridae), in Central Ethiopia. Revue De Medecine Veterinaire. 2000;151 2:143-50.
28. Gabaj M, Beesley W, Awan M. Oestrus ovis myiasis in Libyan sheep and goats. Tropical Animal Health and Production. 1993;25 2:65-8.
29. Pandey V, Ouhelli H. Epidemiology of Oestrus ovis infection of sheep in Morocco. Tropical animal health and production. 1984;16 4:246-52.
30. Oniye S, Adebote D, Ahunanya C. Observations on Oestrus ovis L.(Diptera: Oestridae) myiasis in the Nasal cavities and sinuses of the domestic sheep (Ovis aries) in Zaria, Northern Nigeria. International Journal of Zoological Research. 2006;2 2:178-85.
31. Horak I. Parasites of domestic and wild animals in South Africa. XLVI. Oestrid fly larvae of sheep, goats, springbok and black wildebeest in the Eastern Cape Province. Onderstepoort Journal of Veterinary Research. 2005;72 4:315-20.
32. Pandey V. Epidemiology of Oestrus ovis infection of sheep in the highveld of Zimbabwe. Veterinary Parasitology. 1989;31 3-4:275-80.
33. Horak IG, Butt M. Parasites of domestic and wild animals in South Africa. II. Oestrus ovis in goats. 1977.
34. Saleem T, Katoch R, Yadav A, Mir IA, Godara R, Ahamed I. Prevalence of ovine oestrosis in plain and kandi areas of Jammu, North India. International Journal of Science, Environment. 6 2:1117-22.
35. Sharma M, Gupta A, Mahajan C, Ingle S. Incidence of Oestrus ovis in sheep in Udaipur region of Rajasthan. Veterinary Practitioner. 2012;13(2):360-61
36. Jagannath M, Cozab N, Rahman SA, Honnappa T. Serodiagnosis of Oestrus ovis infestation in sheep and goats. Indian Journal of Animal Sciences. 1989;59 10:1220-4.
37. Pathak K. Incidence of Oestrus ovis in sheep and goats in Rajasthan state of India. Indian Journal of Animal Sciences. 1992;62 1:50-55.
38. Tavassoli M, Tajik H, Malekifard F, Soleimanzadeh A, Mardani K. Seasonal infestation of Oestrus ovis larvae in slaughtered sheep in Urmia, Iran 2012;7 4:98-104.
39. Abo-Shehada MN, Arab B, Mekbel R, Williams D, Torgerson P. Age and seasonal variations in the prevalence of Oestrus ovis larvae among sheep in northern Jordan. Preventive Veterinary Medicine. 2000;47 3:205-12.
40. Othman RA. Seasonal prevalence of Oestrus ovis in Awassi sheep of north of west Bank (Palestine). IUG Journal of Natural Studies. 2015;17 1.
41. Alikhan M, Al-Ghamdi K, Al-Zahrani FS, Khater EI, Allam AM. Prevalence and Salient Morphological Features of Myiasis Causing Dipteran Flies in Jeddah, Saudi Arabia. Biosciences Biotechnology Research Asia. 2018;15 1:101-9.
42. Hanan BA. Seasonal prevalence of Oestrus ovis L.(Diptera: Oestridae) larvae in infested sheep in Jazan Region, Saudi Arabia. Journal of Parasitology and Vector Biology. 2013;5 5:66-71.
43. Özdal N, Tanritanir P, Ilhan F, Değer S. The prevalence of ovine oestrosis (Oestrus ovis Linnaeus, 1761, Diptera: Oestridae) and risk factors in Eastern Turkey. Veterinarski arhiv. 2016;86 3:323-33.
44. Uslu U, Dik B. Prevalence and intensity of Oestrus ovis in Akkaraman sheep in the Konya region of Turkey. Medical and veterinary entomology. 2006;20 3:347-9.
45. Rahman M, Karim M. Incidence of oestrus ovis in sheep and goats. Indian Journal of Animal Sciences 1989;59 10:1216-9.
46. Huq M. Incidence of sheep nasal bot (Oestrus ovis linn. Diptera: Oestridae) in Black Bengal goats [of Bangladesh]. Bangladesh Veterinary Journal (Bangladesh). 1983.
47. Jumde P, Dixit M. Survey of Oesrtus ovis infestation in goats: An abatoir study. Indian Journal of Field Veterinarians. 2012;7 4.
48. Abo-Shehada MN, Batainah T, Abuharfeil N, Torgerson P. Oestrus ovis larval myiasis among goats in northern Jordan. Preventive veterinary medicine. 2003;59 1-2:13-9.
49. Yilma J, Dorchies P. Epidemiology of Oestrus ovis in southwest France. Veterinary Parasitology. 1991;40 3-4:315-23.
50. Bauer C, Steng G, Prevot F, Dorchies P. Seroprevalence of Oestrus ovis infection in sheep in southwestern Germany. Veterinary parasitology. 2002;110 1-2:137-43.
51. Papadopoulos E, Chaligiannis I, Morgan ER. Epidemiology of Oestrus ovis L.(Diptera: Oestridae) larvae in sheep and goats in Greece. Small Ruminant Research. 2010;89 1:51-6.
52. Caracappa S, Rilli S, Zanghi P, Di Marco V, Dorchies P. Epidemiology of ovine oestrosis (Oestrus ovis Linne 1761, Diptera: oestridae) in Sicily. Veterinary Parasitology. 2000;92 3:233-7.
53. Scala A, Solinas G, Citterio C, Kramer L, Genchi C. Sheep oestrosis (Oestrus ovis Linné 1761, Diptera: Oestridae) in Sardinia, Italy. Veterinary Parasitology. 2001;102 1-2:133-41.
54. Scala A, Paz-Silva A, Suárez J, López C, Díaz P, Diez-Banos P, et al. Chronobiology of Oestrus ovis (Diptera: Oestridae) in Sardinia, Italy: guidelines to chemoprophylaxis. Journal of medical entomology. 2002;39 4:652-7.
55. Cozma V, Cernea C, Achelăriţei D, Losson B. The pathology of Oestrus ovis and an investigation on the use of a skin hypersensitivity test for the diagnosis of sheep oestrosis. Sci Parasitol. 2010;11:35e7.
56. Alcaide M, Reina D, Frontera E, Navarrete I. Analysis of larval antigens of Oestrus ovis for the diagnosis of oestrosis by enzyme‐linked immunosorbent assay. Medical and veterinary entomology. 2005;19 2:151-7.
57. Alcaide M, Reina D, Sánchez-López J, Frontera E, Navarrete I. Seroprevalence of Oestrus ovis (Diptera, Oestridae) infestation and associated risk factors in ovine livestock from southwestern Spain. Journal of medical entomology. 2005;42 3:327-31.
58. Gracia MJ, Lucientes J, Peribáñez MA, Castillo JA, Calvete C, Ferrer LM. Epidemiology of Oestrus ovis infection of sheep in northeast Spain (mid-Ebro Valley). Tropical animal health and production. 2010;42 5:811-3.
59. Gracia M, Lucientes J, Peribáñez M, Calvete C, Ferrer L, Castillo J. Kinetics of Oestrus ovis infection and activity of adult flies. Parasite. 2006;13 4:311-3.
60. Paredes-Esquivel C, Monerris M, Martķ T, Borrąs D, Miranda M. High prevalence of myiasis by Oestrus ovis in the Balearic Islands. Parasite. 2009;16 4:323-4.
61. Paredes-Esquivel C, del Rio R, Monerris M, Borràs D, Laglera LM, Miranda MÁ. The influence of sheep age group on the seasonal prevalence of oestrosis in the island of Majorca. Veterinary parasitology. 2012;186 3-4:538-41.
62. Alcaide M, Reina D, Frontera E, Navarrete I. Epidemiology of Oestrus ovis (Linneo, 1761) infestation in goats in Spain. Veterinary parasitology. 2005;130 3-4:277-84.
63. Silva BFD, Machado GP, Izidoro TB, Amarante AFTd. Prevalence of Oestrus ovis (Diptera: Oestridae) in sheep from the São Paulo Central region, Brazil. Revista Brasileira de Parasitologia Veterinária. 2013;22 1:18-21.
64. Silva BFd, Bassetto CC, Amarante AFTd. Epidemiology of Oestrus ovis (Diptera: Oestridae) in sheep in Botucatu, State of São Paulo. Revista Brasileira de Parasitologia Veterinária. 2012;21 4:386-90.
65. Hidalgo A, Palma H, Oberg C, Fonseca-Salamanca F. Oestrus ovis infection of grazing sheep during summer in southern Chile. Pesquisa Veterinária Brasileira. 2015;35 6:497-500.
66. Murguı́a M, Rodrıguez J, Torres F, Segura J. Detection of Oestrus ovis and associated risk factors in sheep from the central region of Yucatan, Mexico. Veterinary parasitology. 2000;88 1-2:73-8.
