# Supplementary material for: The global and regional prevalence of oestrosis in sheep and goats: a systematic review of articles and meta-analysis
Source: Parasit Vectors. 2019 Jul 12;12:346. doi: 10.1186/s13071-019-3597-2 (PMC6625052; doi:10.1186/s13071-019-3597-2)
Supplement: Supplementary file 5 — Additional file 5: Figure S2. Sub-group analysis of ambient environmental temperature at which the peak prevalence of oestrosis was found in sheep and goats in different continents and countries of the world. [file 13071_2019_3597_MOESM5_ESM.docx]

**Additional file 5: Figure S2.** Sub-group of ambient environmental temperature at which the peak prevalence of Oestrosis found in sheep and goats at different continents and countries of the world
